# Trans-eyelid distribution of epinastine to the conjunctiva following eyelid application in rabbits

**DOI:** 10.1007/s10384-024-01070-6

**Published:** 2024-05-25

**Authors:** Takaharu Mochizuki, Tatsuya Hata, Naoto Mori, Takashi Yamazaki, Takahisa Noto, Hidetoshi Mano

**Affiliations:** Pharmaceutics and Pharmacology Department, Nara Research and Development Center, Santen Pharmaceutical Co., Ltd., 8916-16, Takayama-cho, Ikoma-shi, Nara, 630-0101 Japan

**Keywords:** Epinastine, Eyelid, Ocular distribution, Imaging, Allergic conjunctivitis

## Abstract

**Purpose:**

To reveal the penetration of epinastine, an anti-allergic ophthalmic agent, into the eyelid and its distribution to the conjunctiva after administration of a cream formulation on rabbit eyelid skin.

**Study design:**

Experimental study.

**Methods:**

Rabbits were treated with 0.5% epinastine cream on hair-shaved eyelids, followed by preparation of eyelid tissue slices to determine spatial tissue distribution of epinastine by liquid chromatography-tandem mass spectrometry (LC-MS/MS) quantification using laser-microdissected tissues and desorption electrospray ionization mass spectrometry imaging (DESI-MSI). In addition, following either eyelid application of 0.5% epinastine cream or ocular instillation of 0.1% epinastine eye drops, concentration-time profiles of epinastine in the palpebral conjunctiva and bulbar conjunctiva were determined using LC-MS/MS.

**Results:**

Laser microdissection coupled with LC-MS/MS analysis detected high concentrations of epinastine around the outermost layer of the eyelid at 0.5 h post-administration that gradually diffused deeper into the eyelid and was distributed in the conjunctival layer at 8 and 24 h post-administration. Similar time-dependent drug distribution was observed in high-spatial-resolution images obtained using DESI-MSI. Epinastine concentrations in the conjunctival tissues peaked at 4–8 h after administration of 0.5% epinastine cream and then decreased slowly over 72 h post-administration. In contrast, epinastine concentrations peaked quickly and decreased sharply after epinastine eye drop administration.

**Conclusion:**

After the application of epinastine cream to the eyelid skin, epinastine gradually permeated the eyelid. The compound was retained in the conjunctiva for 8–24 h post-administration, indicating that epinastine cream is a promising long-acting formulation for treating allergic conjunctivitis.

**Supplementary Information:**

The online version contains supplementary material available at 10.1007/s10384-024-01070-6.

## Introduction

Topical administration of anti-allergic eye drops is the first-line treatment for treating allergic conjunctivitis, one of the common inflammatory diseases in the conjunctiva causing ocular itching, redness, and swelling [1, 2]. However, eye drops are rapidly eliminated from the ocular surface after administration by blinking, tear turnover, and flow through the nasolacrimal duct [3, 4], causing rapid elimination of anti-allergic drugs from the ocular tissues. Owing to this, most anti-allergic eye drops require two to four applications a day [5, 6]. Multiple administration of eye drops typically imposes a burden on patients, leading to poor treatment compliance and outcomes. In the USA, anti-allergic eye drops allowing for once-daily instillation have been approved [7]; however, their anti-allergic effects weaken with time [8–11]. To sustain maximum anti-allergic effects with reduced dosing frequency, several drug delivery techniques have been investigated [12–14].

Recently, drug delivery through the eyelid has been suggested as an effective dosing route for treating allergic conjunctivitis [15–17]. When epinastine, an antihistaminic agent that acts as a histamine H1 receptor blocker and mast cell stabilizer, was applied to the eyelid skin of guinea pigs with histamine-induced conjunctivitis, elevated conjunctival vascular permeability was strongly inhibited at 24 h post-administration, significantly greater than after ocular instillation of commercially available 0.1% epinastine eye drop. In addition, after eyelid application of epinastine, the maximum inhibitory effect was observed at 2 h post-administration and then sustained over 24 h post-administration [17]. These findings strongly indicate that eyelid application of epinastine can be a promising approach for once-daily dosing with sustained efficacy for treating allergic conjunctivitis.

Once a drug partitions into the body’s skin, it diffuses slowly by passive diffusion in the stratum corneum, the outermost layer of the skin, and further penetrates the deeper epidermis and dermis layers [18, 19]. This behavior causes a slower peak of tissue concentration and retention of effective concentration in the tissues than by other dosage options, such as oral and intravenous administration [20, 21]. Thus, drug application to the eyelid is assumed to show sustained drug concentrations in the inner layer of the eyelid and adjacent ocular tissues compared to ocular instillation with eyedrops. However, the eyelid has a unique tissue composition, unlike the arm and abdominal skin [22]. The number of stratum corneum cell layers in the eyelid is 8 ± 2, fewer than in the abdominal skin (14 ± 2) [23]. When moving from the epidermis and dermis to the deeper eyelid, the orbicularis oculi muscle lies over the inner lamellae, including the orbital septum, tarsal plate, and levator palpebrae superioris muscle [24, 25]. The palpebral conjunctiva is found at a deeper area of the eyelid and underlies the tarsal plate and Müller muscle [26]. These structural differences between the eyelid and the skin of other body parts imply that the drug penetration pattern in the eyelid may be different from those elucidated in prior research using arms and abdominal skin. However, to our knowledge, little research has been conducted to reveal how the administered chemical entities penetrate the eyelid and are distributed in the inner tissues.

In this study, we investigated the distribution of epinastine to the conjunctiva, the pharmacological target of epinastine in allergic conjunctivitis, after the application of an epinastine cream formulation to rabbit eyelid skin. To disclose drug penetration behavior from the epidermis to the conjunctiva with visualized images, we employed spatial distribution analysis in the eyelid using laser microdissection (LMD) coupled with liquid chromatography-tandem mass spectrometry (LC-MS/MS) and mass spectrometry imaging (MSI) that have been generally used to evaluate spatial distribution in several tissues [27–30]. In addition, to characterize the pharmacokinetics of epinastine in the conjunctival tissues after eyelid application, we assessed concentration-time profiles of epinastine in the tissues and compared their behavior with that after ocular instillation of eye drops.

## Materials and methods

This study followed the Association for Research in Vision and Ophthalmology (ARVO) Statement for the Use of Animals in Ophthalmic and Vision Research. It was approved and monitored by the Institutional Animal Care and Use Committee of Santen Pharmaceutical Co., Ltd.

### Drug treatment procedure for rabbit eyelid skin

Before treatment, Japanese white rabbits (3–4 kg; Kitayama Labes) were anesthetized with ketamine (Ketalar; Daiichi Sankyo) and xylazine (Selactar; Bayer) at doses of 21.9 and 1.3 mg/kg, respectively, and the hairs on their upper and lower eyelids were removed using an electric razor. A water in oil (W/O) type cream containing 0.5% epinastine hydrochloride, obtained from a contract manufacturing organization, was applied to the upper and lower eyelids by gentle spreading using a pipette tip. Because 0.5% epinastine was the most effective concentration in histamine- and ovalbumin-induced allergic conjunctivitis guinea pig models in previous pharmacological experiments [17], it was used in this study as well. The application site was set 5 mm from the lid margin to prevent the cream from entering the eye directly (Online Resource 1). On each eyelid, 10 µL of cream was applied to an approximately 0.7 cm^2^ area for spatial distribution analysis and 15 µL of cream was applied to an approximately 1 cm^2^ area for pharmacokinetics evaluation. The dosing volume was reduced from the assumed clinical dosing volume (15 µL) in the spatial distribution analysis to clearly determine how the cream spread both into the deeper eyelid and on the eyelid surface. An Elizabethan collar was placed around the neck of each rabbit until tissue collection.

### Preparation of eyelid tissue section for spatial distribution analysis

At 0.5, 8, and 24 h after eyelid treatment, the rabbits (one rabbit per time point) were euthanized by an intravenous injection of pentobarbital sodium (64.8 mg/mL) at a dose of 2 mL/kg. The eyelid of each animal was harvested and immediately frozen on dry ice, followed by sectioning (thickness: 10 μm) with a CM3050S cryostat (Leica Microsystems). The sections were mounted on a glass slide and stored at − 80 °C until LMD-LC-MS/MS and MSI analyses.

### LMD sample processing and quantitation of epinastine

The eyelid tissue sections were dissected using an LMD7 laser microdissection system (Leica Microsystems). The area of interest was visualized under a microscope, and a line was drawn around the target specimen for cutting. The target surface area displayed in the software was recorded, and the region was dissected. Each dissected portion was collected in a 0.5 mL PCR tube (Corning). Dissected samples were associated with their corresponding surface areas. Each dissected sample was homogenized and mixed with an internal standard (epinastine-^13^C, d_3_ hydrobromide; Medical Isotopes) solution. The mixture was cleaned using an Oasis HLB 96-well µElution Plate (Waters). Calibration standards and quality control samples were prepared using a standard solution (epinastine hydrochloride; Boehringer Ingelheim) for each batch of analyses. The processed samples were injected into Shimadzu Nexera HPLC (Shimadzu) coupled with a QTRAP7500 tandem mass spectrometer equipped with an electrospray ionization source (Sciex). Chromatographic separation was performed using a Kinetex XB-C18 (50 mm × 2.1 mm i.d., 2.6 μm) analytical column (Phenomenex). The mobile phase used for the analysis was 0.2% formic acid and 17% acetonitrile. The flow rate was set to 0.35 mL/min. The mass spectrometer was operated in the multiple reaction monitoring mode with positive polarity by monitoring the precursor and product ions at m/z 250 and 208, respectively, for epinastine and m/z 254 and 212, respectively, for epinastine-^13^C, d_3_. Data acquisition and quantitative analysis were conducted using SCIEX OS 2.2 software (Sciex). The epinastine concentration in each dissected portion was normalized to its corresponding volume. The volume of the dissected portion was calculated by multiplying the surface area by the thickness (10 μm).

### Desorption electrospray ionization (DESI) mass imaging

DESI-MSI data of the sections on glass slides were acquired using a DESI source (Waters) equipped with a quadrupole time-of-flight mass spectrometer (Xevo G2-XS QTof; Waters) in the positive ion mode. Before the analysis, the DESI-MSI instrument was calibrated using a 10 mM sodium formate solution prepared in 50% methanol. An ACQUITY UPLC binary solvent manager (Waters) was used to deliver 98% methanol as DESI spray solvent at a speed of 3 µL/min. The capillary and cone voltages were set to 3.5 kV and 50 V, respectively. The source temperature was set at 120 °C. Mass spectra were acquired in the m/z 100–1,000 range. The pixel size was 100 μm. The mass resolution was set at 20,000. The Mass Lynx 4.1 and HD Imaging 1.4 software (Waters) were used to obtain the DESI-MSI data. A distribution image of the upper eyelid sections was created using protonated epinastine ions at m/z 250.13 without normalization.

### Pharmacokinetic evaluation in ocular tissues

At 2, 4, 8, 24, and 72 h after eyelid treatment to both eyes, the rabbits (two rabbits per time point) were euthanized by intravenous injection of pentobarbital sodium (64.8 mg/mL) at a dose of 2 mL/kg, followed by harvesting the palpebral conjunctiva and bulbar conjunctiva.

For ocular instillation, eye drops containing 0.1% epinastine hydrochloride (Santen Pharmaceutical), were used, the concentration of the market approved product. At 0.25, 0.5, 1, 2, 4, 8, and 12 h after administration of 50 µL of eye drops, the rabbits (two rabbits per time point) were euthanized by intravenous injection of pentobarbital sodium (64.8 mg/mL) at a dose of 2 mL/kg, followed by harvesting the palpebral conjunctiva and bulbar conjunctiva.

The conjunctival tissues were homogenized and mixed with the internal standard (epinastine-^13^C, d_3_ hydrobromide) solution. The mixture was cleaned using an Oasis HLB 96-well µElution Plate. Calibration standards and quality control samples were prepared using the standard (epinastine hydrochloride) solution for each batch of analyses. The processed samples were injected into a Shimadzu Nexera HPLC coupled with a QTRAP6500 + tandem mass spectrometer equipped with an electrospray ionization source (Sciex). Chromatographic separation was performed using a Kinetex XB-C18 (50 mm × 2.1 mm i.d., 2.6 μm) analytical column. A gradient of 0.1% formic acid in water and acetonitrile was applied at a constant flow rate of 0.35 mL/min. The mass spectrometer was operated in the multiple reaction monitoring mode with positive polarity by monitoring the precursor and product ions at m/z 250 and 193, respectively, for epinastine and m/z 254 and 195, respectively, for epinastine-^13^C, d_3_. Data acquisition and quantitative analysis were conducted using the Analyst 1.7.1 software (Sciex).

## Results

### Determination of epinastine penetration into rabbit eyelids using laser-microdissection and LC-MS/MS

**Fig. 1:**
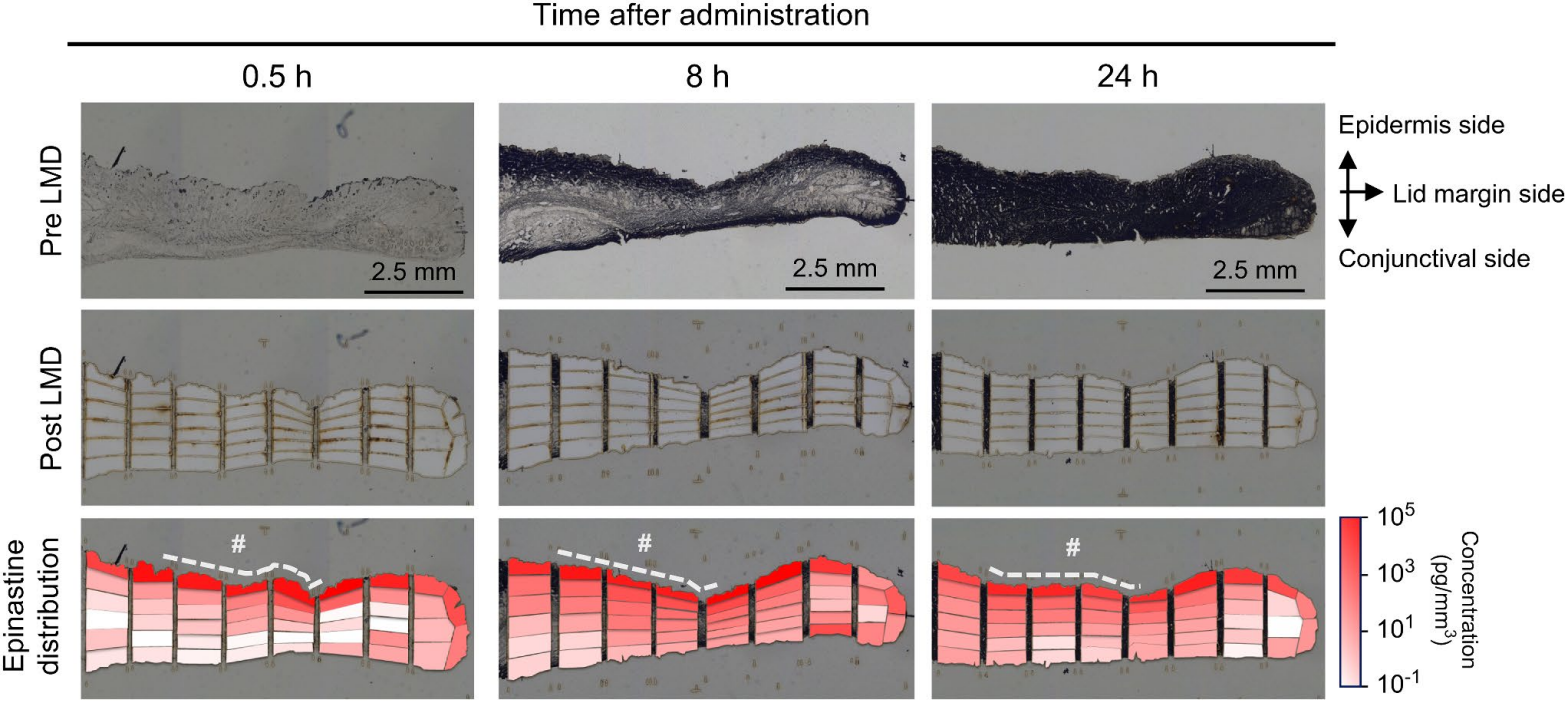
Determination of epinastine concentrations in rabbit upper eyelid compartments using laser-microdissection (LMD) coupled with liquid chromatography-tandem mass spectrometry (LC-MS/MS) Optical images of rabbit upper eyelid frozen sections before laser-microdissection (LMD) (“Pre LMD”) and after LMD (“Post LMD”), and epinastine concentrations in microdissected fragments (“Epinastine distribution”) at 0.5, 8, and 24 h after eyelid application of 0.5% epinastine cream. Compartments where the epinastine concentrations were below the lower limit of quantification (< 0.1 pg/mm^3^) are shown in white. #: Approximate area for cream application

To reveal how epinastine is distributed in the eyelid after topical administration on the eyelid skin, we prepared tissue sections from rabbit eyelids collected at 0.5, 8, and 24 h after administration (Fig. 1, Pre LMD), followed by the dissection of approximately 20,000–50,000 µm^2^ of compartments from the eyelid sections using LMD (Fig. 1, Post LMD). Epinastine concentrations in the microdissected fragments were determined using highly sensitive LC-MS/MS, as shown in Fig. 1 (“Epinastine distribution”). At 0.5 h after administration, 2.0–4.0 × 10^4^ pg/mm^3^ of epinastine was observed in microdissected fragments on the uppermost layer of the eyelid section, while > 10-fold lower concentrations were observed when moving to deeper sections. Large parts of the microdissected fragments in the inner eyelid and conjunctival side exhibited < 10 pg/mm^3^ of epinastine. In the uppermost layer of the tissue slice, relatively high concentrations were observed toward the marginal end of the eyelid, where the epinastine cream was not applied, indicating that the cream was spread on the eyelid surface. At 8 and 24 h post-dose, epinastine concentrations in the deeper parts of the eyelid sections and conjunctival layer increased compared to those at 0.5 h post-dose, and were relatively uniform across those areas, except for the area close to the lid margin in the sample at 24 h post-dose. High epinastine concentrations (2.0 × 10^3^–2.0 × 10^4^ pg/mm^3^) remained in the uppermost layer of the eyelid at 24 h after eyelid application.

### Determination of spatial distribution images of epinastine in rabbit eyelid sections using DESI-MSI

**Fig. 2:**
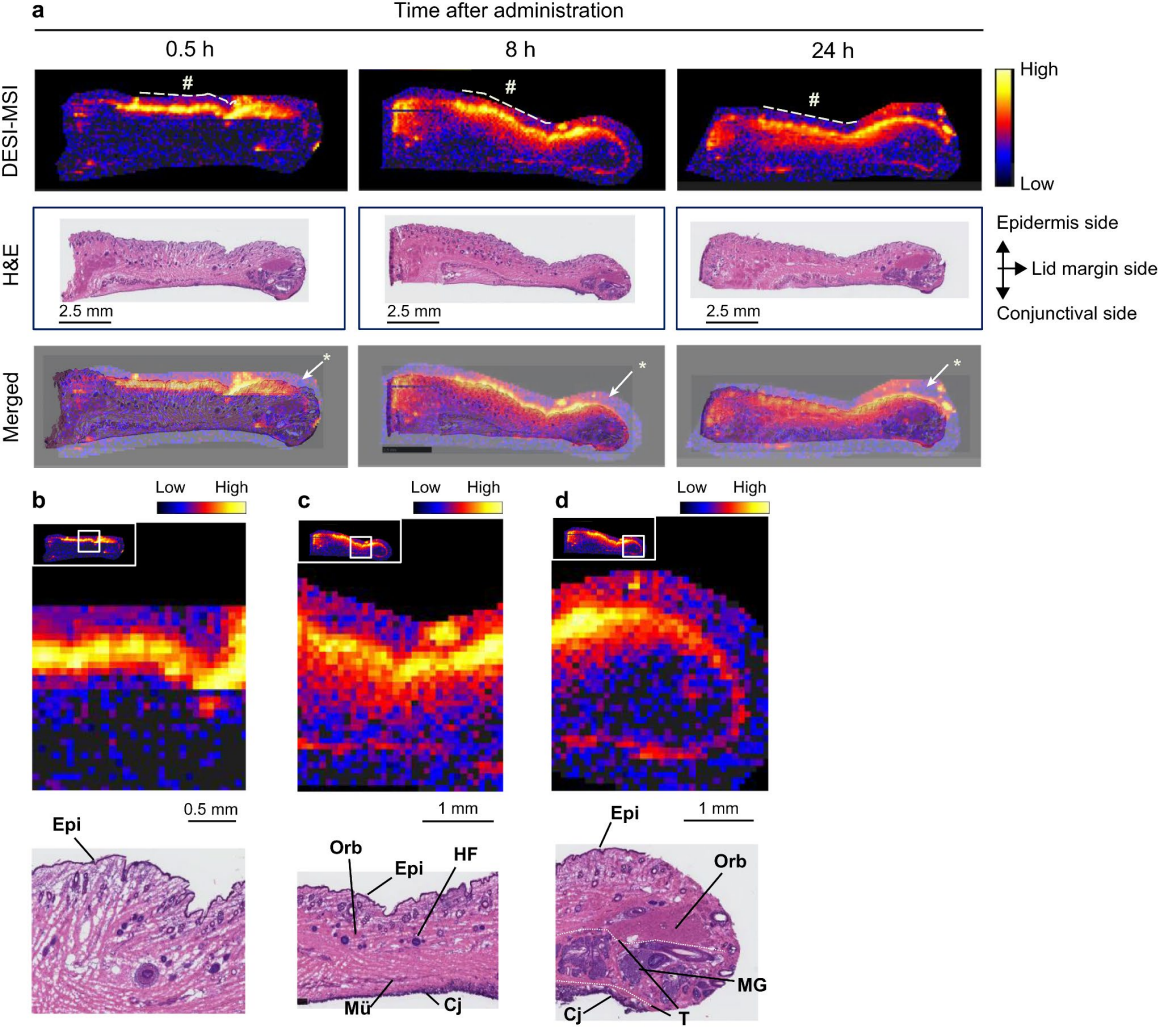
Imaging of epinastine distribution in rabbit upper eyelid section obtained using desorption electrospray ionization mass spectrometry imaging (DESI-MSI) a Whole distribution images of epinastine in rabbit upper eyelid sections determined by DESI-MSI, optical images of hematoxylin-eosin (H&E) staining and merged images (Merged) at 0.5, 8, and 24 h after eyelid application of 0.5% epinastine cream. #: Approximate area for cream application; *: Artifact signals surrounding eyelid tissue. b–d Magnified images of DESI-MSI (upper) and H&E (lower) at 0.5 h (b) and 8 h (c and d) post-dose. d The connective tissues surrounding the meibomian gland indicate the tarsal plate. *Epi*, epidermis; *Orb*, orbicularis oculi muscle; *HF*, hair follicle; *Mü*, Müller muscle; *Cj*, conjunctival epithelium; *MG*, meibomian gland; *T*, tarsal plate

DESI-MSI analysis was performed using serial eyelid sections from those used in the LMD-LC-MS/MS experiments to demonstrate epinastine distribution with higher spatial resolution images. Figure 2a shows whole-eyelid images obtained using DESI-MSI and hematoxylin-eosin (H&E) staining. H&E staining was performed after DESI-MSI using the same tissue slices. At 0.5 h post-dose, a higher intensity was observed in the uppermost layer while a lower intensity was observed in the inner eyelid and conjunctival areas. At 8 and 24 h after administration, surface signals diffused into the inner part of the eyelid, and a signal intensity distribution was found in the conjunctival layers. A strong intensity was still detected around the epidermis at 24 h post-dose. Consistent with the LMD-LC-MS/MS experiment observations, a strong intensity around the outermost layer beyond the cream application site was observed in all samples.

The magnified image at 0.5 h shows high signal intensity mainly around the epidermis and upper dermis (Fig. 2b), while medium levels of signal intensity were observed over the region where the orbicularis oculi muscles and hair follicles were located, with unchanged higher signals in the epidermis and upper dermis areas, at 8 h post-administration (Fig. 2c). At the marginal end of the eyelid in the section at 8 h post-dose, signals were detected in the dermis area. In contrast, the intensity was negligible, where the orbicularis oculi muscle fibers and tarsal plate, including the meibomian gland, were localized (Fig. 2d).

### Pharmacokinetics in rabbit conjunctiva after administration of epinastine cream and eye drops

**Fig. 3:**
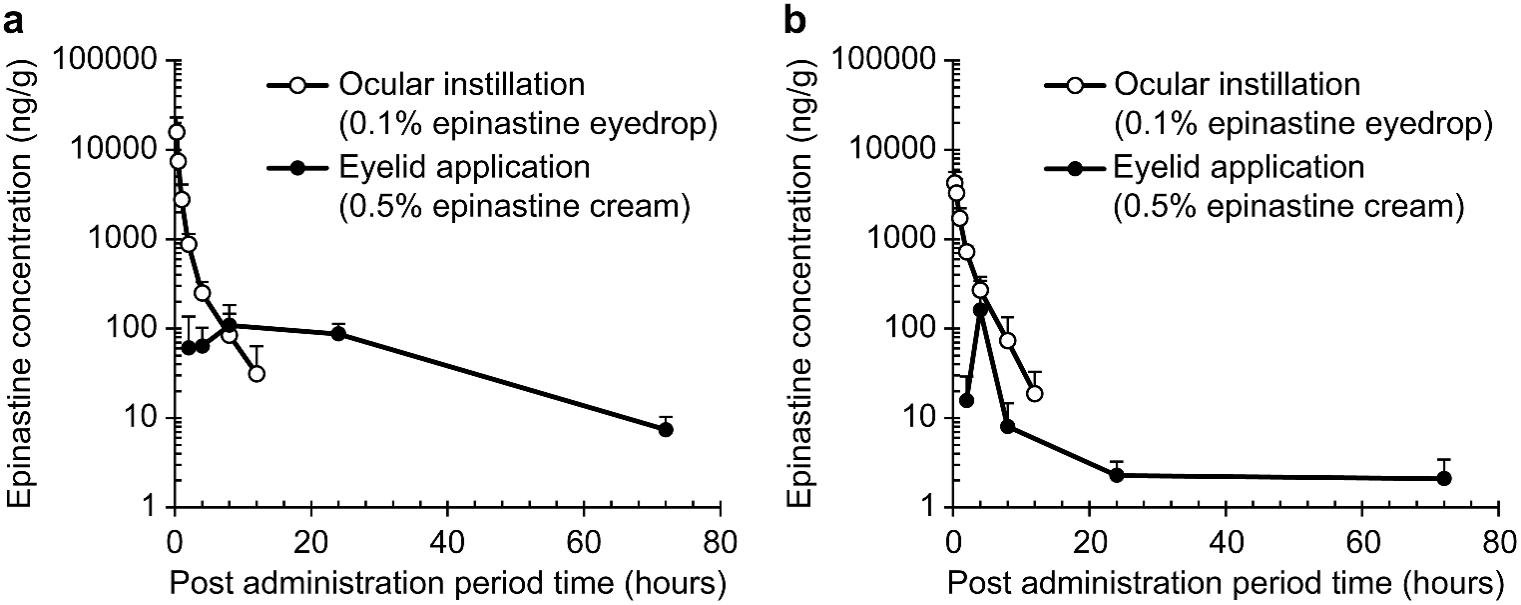
Epinastine concentrations in the conjunctiva after topical eyelid application of 0.5% epinastine cream and ocular instillation of 0.1% epinastine eye drops in rabbits Each value represents the mean and standard deviation of four eyes. Palpebral conjunctiva (**a**) and bulbar conjunctiva (**b**)

To characterize the drug pharmacokinetics in the target tissue after eyelid application, epinastine concentrations in the palpebral conjunctiva and bulbar conjunctiva were determined using LC-MS/MS. Figure 3 shows the concentration-time profile of epinastine after administration of 0.5% epinastine cream (150 µg epinastine/eye) on the eyelid skin and 0.1% epinastine eye drops (50 µg epinastine/eye) in the eye. After applying the cream to the eyelid, the concentration of epinastine in the palpebral conjunctiva peaked at 8 h, followed by a gradual decrease over 72 h after administration. At 24 h after cream administration, the epinastine concentration was 87.3 ng/g, approximately 80% of the peak concentration (109 ng/g). In contrast, after eye drop administration, epinastine concentrations in the palpebral conjunctiva were 15,800 ng/g at 0.25 h, then decreased rapidly to 31 ng/g at 12 h post-dose (Fig. 3a). In the bulbar conjunctiva, the concentration was 162 ng/g at 4 h after eyelid application, then decreased to 8.1 ng/g at 8 h post-administration. The concentration was relatively retained for 8–72 h after administration. Epinastine concentration in the bulbar conjunctiva decreased quickly after eye drop administration as observed in the palpebral conjunctiva (Fig. 3b).

## Discussion

Recently, drug application to the eyelid has been highlighted as a new route for treating ocular disorders [15–17]. In this study, we investigated how epinastine, an ophthalmic agent used to treat ocular allergic diseases, penetrates the eyelid, and is distributed to the conjunctiva when a cream formulation was applied to rabbit eyelid. In general, drug penetration into deeper skin areas is restricted by the stratum corneum barrier, and subsequently, a small amount of drug can be distributed into the inner area [18, 31]. Therefore, highly sensitive quantification techniques are required to determine spatial distribution in the eyelid. Radiolabeled compounds are typically used to assess spatial drug distribution in tissues [32]; however, this experimental approach has some technical limitations such as the inability to evaluate drugs and their metabolites separately [33]. To overcome these difficulties, we used an LMD-LC-MS/MS method that allowed the detection of low amounts of drugs in the tissues without the need for compound labeling. Using this technique, we found that epinastine diffused into the eyelid over time and was distributed to the palpebral conjunctiva after application of epinastine cream to the eyelid skin (Fig. 1). To the best of our knowledge, this is the first report to disclose the detailed spatial drug distribution in the eyelid after application to the eyelid. The distribution image at 0.5 h post-dose demonstrates that epinastine concentrations dramatically declined when moving from the outermost layer to the deeper eyelid section (Fig. 1). This visualized image confirmed that the stratum corneum in the eyelid skin acted as a barrier to epinastine penetration, although these stratum corneum layers are thinner than those in the skin in other areas of the body [23]. At 8 h post-dose, epinastine was distributed more uniformly in the eyelid and reached the conjunctiva in the opposite region of the eyelid (Fig. 1), indicating that epinastine can penetrate the eyelid and be distributed to the conjunctival layer. Similar distribution patterns were observed in lower eyelid sections, indicating that the lower eyelid skin is also an effective dosing route for epinastine treatment (Online Resource 2).

To further elucidate epinastine distribution in the eyelid, DESI-MSI was performed, which can provide high-spatial-resolution images compared to LMD-LC-MS/MS analysis [34]. DESI-MSI demonstrated that epinastine was distributed mainly around the epidermis and upper dermis at 0.5 h post-dose (Fig. 2b). In contrast, medium levels of epinastine diffusion were observed in the inner region, in addition to a higher surface distribution at 8 h after application (Fig. 2c). These DESI-MSI findings also confirmed the barrier and reservoir-like functions of the stratum corneum in the eyelid skin. In contrast to the eyelid surface, in the present study epinastine distribution in the deeper eyelid region, including the area close to the lid margin, was not clearly detectable using DESI-MSI. Because MSI technique detects chemical species only in a spot on the tissue slice, it is disadvantageous for highly sensitive drug quantitation compared to LMD-LC-MS/MS [34]. However, high-spatial-distribution images obtained in this study confirmed a possible drug distribution route to the conjunctiva, which was not clearly revealed by LMD-LC-MS/MS analysis. At 8 h post-dose, epinastine distribution at the lid margin was mainly observed in the epidermis and dermis areas (Fig. 2d), suggesting that epinastine may be distributed to the conjunctiva by detouring the orbicularis muscle and tarsus at the lid margin. To elucidate the detailed drug distribution route in the eyelid, further investigation using a more sensitive MSI technique remain warranted.

High levels of epinastine were observed in the outermost layer beyond the application area (Figs. 1 and 2a). This phenomenon can be attributed to the spread of epinastine cream on the eyelid surface as the viscosity of the cream decreased due to the body temperature, as well as to the blinking and movement of the animals. The spread of cream on the eyelid was observed as early as at 0.5 h post-dose, and this movement likely led to the relatively high concentrations in the conjunctiva around the lid margin at the same time point (Fig. 1 and Online Resource 2). When discussing this drug distribution behavior at early phase, we could not rule out the possibility that the cream directly entered the eye and distributed to the conjunctiva in this study. To address this question, evaluation with both finer time points and high-spatial-resolution images in the early phase could provide a clearer understanding of the drug distribution route to the conjunctiva. Additionally, conducting similar experiments with compounds that do not penetrate the skin (such as macromolecules) will help elucidate the contribution of the route using which the cream directly enters the eye and gets distributed to the ocular tissues.

The pharmacokinetic evaluation demonstrated that the pharmacokinetic pattern of epinastine in the conjunctival tissues differed between eyelid cream application and ocular instillation of epinastine eye drops (Fig. 3a and b). When epinastine cream was administered to the eyelid skin, epinastine concentrations increased with a slow absorption pattern, followed by a slow decrease in the tissue. Meanwhile, after the instillation of eye drops, the highest levels of epinastine were observed at 0.25 h and the compound was eliminated rapidly. Our findings indicate that epinastine applied to the eyelid partitioned to the stratum corneum and diffused slowly into the eyelid, likely due to the reservoir action of the stratum corneum [19]. In a histamine-induced allergic conjunctivitis guinea pig model, elevated vascular permeability in the conjunctiva was strongly inhibited at 2 h after eyelid application of 0.5% epinastine cream, and the maximum inhibitory effect was sustained over 24 h after administration [17]. The longer residence time of epinastine in the conjunctiva found in this study supports the prolonged anti-allergic activity observed in the aforementioned report. Multiple daily dosings of eye drops is challenging for some patients and causes low adherence and negative treatment outcomes [35–37]. Both pharmacology and pharmacokinetic observations using epinastine cream strongly indicate that applying epinastine to the eyelid can be an effective option for treating allergic conjunctivitis by maintaining strong anti-allergic activity following once daily treatment.

In the bulbar conjunctiva, epinastine concentrations at 8–24 h were 2.3–8.1 ng/g, lower than those in the palpebral conjunctiva (87.3–109 ng/g) (Fig. 3b). These findings may be attributed to the fact that the bulbar conjunctiva is located further away from the eyelid skin than the palpebral conjunctiva. Following eyelid application of 0.5% epinastine cream to a guinea pig model of ovalbumin-induced allergic conjunctivitis, improvement in edema and redness were observed in the whole conjunctiva [17], suggesting that sufficient concentrations of epinastine to exhibit an anti-allergic effect were distributed to the bulbar conjunctiva.

In the human eyelid, the thickness of the stratum corneum layer, a rate-limiting barrier in skin penetration of drugs, is approximately 14.9 μm, thicker than in rabbit eyelids (approximately 7.5 μm) [15, 38]; therefore, epinastine penetration into the eyelid can be limited in humans compared with that in rabbits. However, it is plausible that sufficient levels of epinastine can be distributed to the conjunctival tissues in humans following eyelid application since significant repressions of ocular itching and conjunctival hyperemia have been demonstrated in subjects with allergic conjunctivitis after once-daily eyelid application of 0.5% epinastine cream [39].

Investigating pharmacokinetic behavior is essential for predicting the efficacy and safety of candidate drugs during pharmaceutical development. Moreover, as the eyelid has a unique tissue composition, including muscle and connective tissues, determining detailed drug distribution in each tissue compartment may reveal pharmacokinetic-pharmacodynamic and pharmacokinetic-toxicological insights more clearly. We anticipate that mass spectrometry-based spatial distribution approaches such as LMD-LC-MS/MS and MSI will facilitate drug development for eyelid-related diseases such as blepharitis, meibomian gland dysfunction, and acquired ptosis as well as allergic conjunctivitis [40, 41].

In the present study, we assessed epinastine distribution to the conjunctiva using the spatial tissue distribution analysis and conventional tissue collection followed by drug quantitation. After application of epinastine cream to the eyelid skin, epinastine can penetrate the eyelid and distribute to the conjunctiva with longer retention than that achieved with eye drop instillation. These findings suggest that drug delivery of epinastine through the eyelid can be an effective approach for the treatment of allergic conjunctivitis.

## Electronic supplementary material

Below is the link to the electronic supplementary material.

Supplementary Material 1

Supplementary Material 2
