# Supplementary material for: Trans-eyelid distribution of epinastine to the conjunctiva following eyelid application in rabbits
Source: Jpn J Ophthalmol. 2024 May 25;68(5):594–602. doi: 10.1007/s10384-024-01070-6 (PMC11420250; doi:10.1007/s10384-024-01070-6)
Supplement: Supplementary file 1 — Supplementary Material 1 [file 10384_2024_1070_MOESM1_ESM.pdf]

Supplemental figure 1
(Online Resource 1)

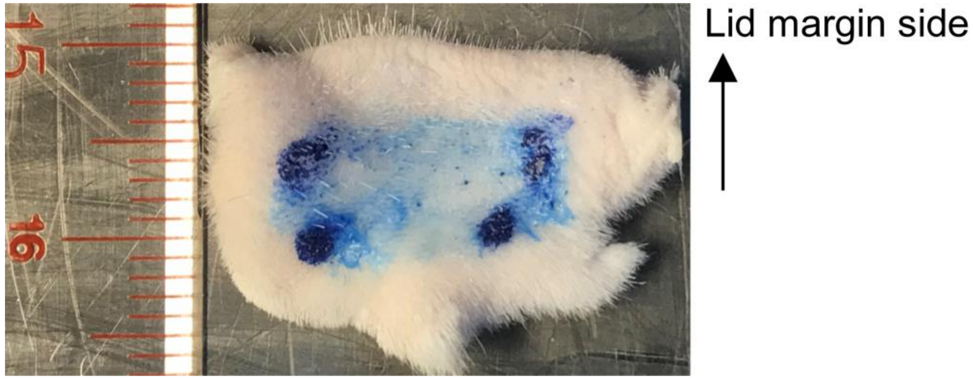

Representative picture of the cream application site on rabbit eyelids
Epinastine cream was applied 5 mm from the lid margin. The area corresponding to the cream application site is depicted in blue
