# Supplementary material for: Trans-eyelid distribution of epinastine to the conjunctiva following eyelid application in rabbits
Source: Jpn J Ophthalmol. 2024 May 25;68(5):594–602. doi: 10.1007/s10384-024-01070-6 (PMC11420250; doi:10.1007/s10384-024-01070-6)
Supplement: Supplementary file 2 — Supplementary Material 2 [file 10384_2024_1070_MOESM2_ESM.pdf]

Supplemental figure 2
(Online Resource 2)

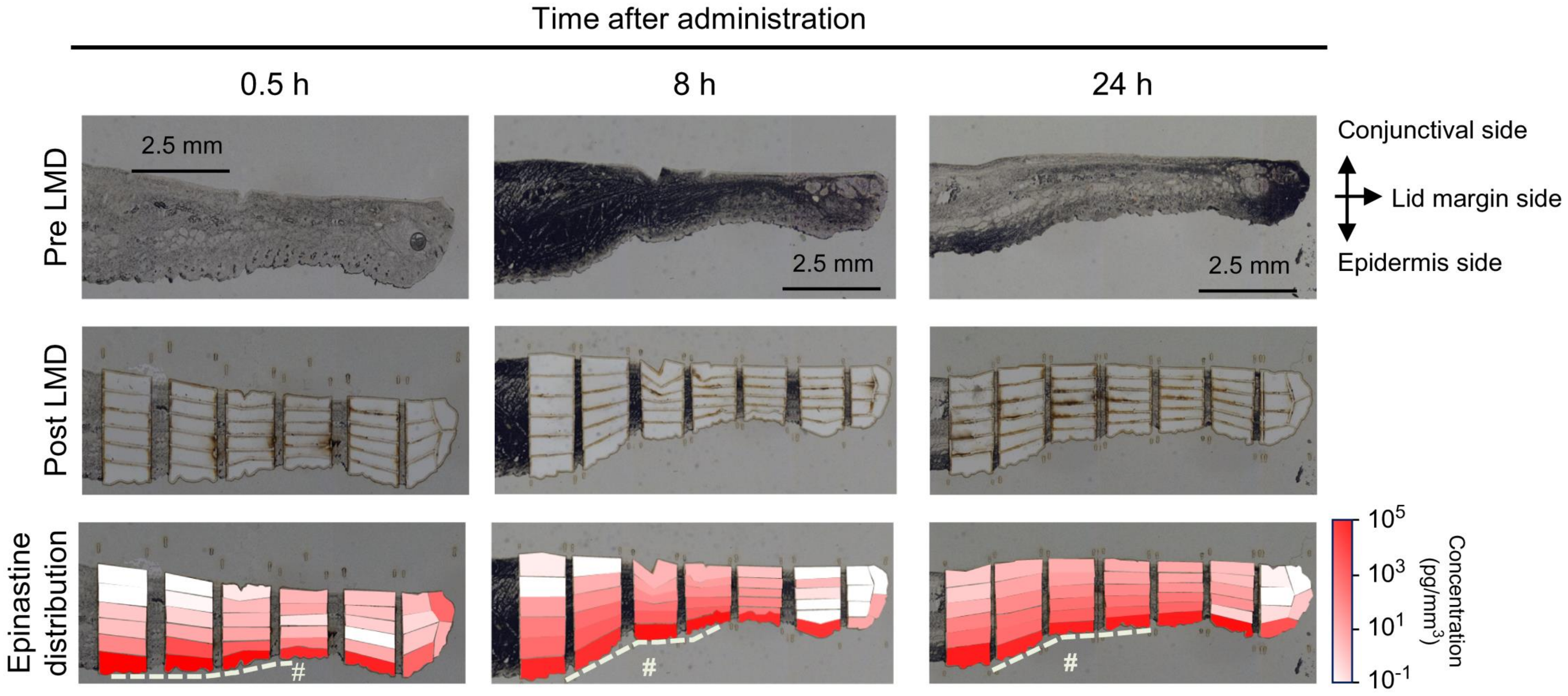

Spatial distribution images of epinastine in rabbit lower eyelid obtained using laser-microdissection (LMD) coupled with liquid chromatography-tandem mass spectrometry (LC-MS/MS)

#: Approximate area for cream application.
